# Clinical Value of Preoperative Ultrasound Signs in Evaluating Axillary Lymph Node Status in Triple-Negative Breast Cancer

**DOI:** 10.1155/2022/2590647

**Published:** 2022-05-14

**Authors:** Jundong Wang, Xiaoli Lu, Xuan Zheng, Congyan Xia, Ping Li

**Affiliations:** Department of Ultrasound, Nanjing First Hospital, Jiangsu Province, China

## Abstract

**Purpose:**

To explore the clinical value of preoperative ultrasound signs in evaluating axillary lymph node status in triple-negative breast cancer (TNBC).

**Methods:**

A retrospective study was conducted on 162 patients with TNBC who were admitted to our hospital from January 2017 to June 2021. A total of 62 patients with axillary lymph node metastasis and 100 patients with normal axillary lymph nodes were included. Univariate and logistic regression was used to analyze the correlation between clinicopathological parameters, ultrasound features, and axillary lymph node metastasis between these two groups. The receiver operating characteristic (ROC) curve of each index was drawn to predict positive axillary lymph node.

**Results:**

The lymph node positive rate was higher in patients with tumor size (2 mm < *T* ≤ 5 mm) and tumor stage III, and the difference between these two groups was statistically significant (*P* < 0.05). The patients with cortical thickness ≥ 3, blood flow grades II-III, aspect ratio (L/S) ≥ 2, and RI ≥ 0.7 had higher lymph node positive rate, and the difference between these two groups was statistically significant (*P* < 0.05). Other index shows no correlation with ancillary lymph node positive rate, or the correlation was not statistically significant (*P* > 0.05). Further regression analysis indicated that the blood flow grade and L/S of axillary lymph nodes were independent influencing factors of axillary lymph node metastasis in TNBC patients (*P* < 0.05). Relevant receiver operating characteristic (ROC) curves were constructed, and the AUC of axillary lymph node blood flow grade and L/S for predicting axillary lymph node status was 0.6329 and 0.6498, respectively. The AUC for the joint prediction of the two indicators is 0.6898.

**Conclusion:**

Ultrasound sign combined with clinicopathological characteristics can predict the axillary lymph nodes metastasis in TNBC, which could guide clinical decision of axillary lymph node surgery.

## 1. Introduction

Breast cancer seriously threatens the health of women. At present, the incidence of breast cancer is increasing every year and in younger population. Some studies have reported that the number of breast cancer in women under the age of 40 years old continues to increase, and about 11% of the new patients are not older than 45 years old, which has become a public health concern [1]. Axillary lymph nodes are the most common metastatic sites of breast cancer, accounting for about 75% of total metastasis of breast cancer. Axillary lymph node metastasis is the most important predictor of recurrence of the tumor and survival rate for the breast cancer patients. Studies have shown that the 5-year survival rate of patients with axillary lymph node metastasis is about 85.8% [2]. For patients with axillary lymph node metastasis, axillary lymph node dissection can improve the survival rate. However, for breast cancer patients with no axillary lymph node metastasis confirmed by postoperative pathological results, axillary lymph node dissection has no therapeutic effect. Therefore, accurate preoperative assessment of axillary lymph node status is important for breast cancer staging and clinical decisions for appropriate treatment.

Currently, almost all breast cancer patients undergo sentinel node biopsy to determine the status of the axillary lymph nodes. However, studies have demonstrated that more than 60% of patients with early-stage breast cancer are confirmed to be node-negative via pathological examination who are not benefit from sentinel lymph node biopsy. Therefore, whether a noninvasive assessment method of axillary lymph node status can replace sentinel node biopsy or fine-needle aspiration biopsy (FNAC) increasing attention, preoperative axillary ultrasound is routinely used in clinical practice, and metastatic axillary lymph nodes have certain characteristics on ultrasound images. However, guidelines, consensus, and diagnostic criteria for assessing the status of axillary lymph nodes using ultrasound are inadequate, which makes the results of ultrasound assessment of axillary lymph nodes controversial. Moreover, the differences in operator experience and the resolution of ultrasound instruments reduced the accuracy of ultrasound in detecting metastatic lymph node. Several studies are also currently investigating whether axillary ultrasonography can replace sentinel point biopsy in patients with CT1N0 breast cancer [3, 4].

Triple-negative breast cancer (TNBC) is the most aggressive subtype of breast tumors and lacks estrogen receptors, progesterone receptors, and human epidermal growth factor receptors [5, 6]. TNBC accounts for 15% to 20% of all breast cancer incidences. Compared with other breast cancer subtypes, TNBC usually has larger mass, higher lymph node positive rate, aggressiveness, recurrence rate, and poor prognosis [7, 8]. At present, there are few studies on the influencing factors of ultrasound on axillary lymph node metastasis of TNBC. This article is aimed at exploring the influencing factors of ultrasound in diagnosing lymph node metastasis in TNBC, predicting the metastasis of axillary lymph nodes, and selecting the surgical plan for axillary lymph nodes.

## 2. Material and Method

### 2.1. Study Subjects

This is a retrospective study on 162 TNBC patients who were admitted to Nanjing First Hospital from January 2017 to June 2021, aged 27-78 years, with an average of 48.39 ± 10.83 years old. All enrolled patients signed informed consents. The patients' demographics are shown in Table 1. Inclusion criteria were as follows: (1) female patients diagnosed with breast cancer by surgery and pathology; (2) patients with negative ER, PR, and HER-2 in postoperative immunohistochemical or fluorescence in situ hybridization (FISH) examinations; and (3) patients with clear preoperative ultrasound images and complete data. Exclusion criteria were as follows: (1) patients who received breast cancer surgery, chemotherapy, radiotherapy, and other treatments before surgery and (2) patients who did not sign the informed consent form or did not agree to be enrolled.

## 3. Methods of Examination

### 3.1. Ultrasound Examination

The color Doppler ultrasound diagnostic instrument used for ultrasound examination is MyLab Twice (Esaote Company, Italy). The frequency of the linear array probe was 4-13 MHz. The ultrasound images of the patients were reviewed and analyzed by 2 experienced sonographers. If difference in diagnosis occurred, an agreement was reached after discussion. All patients were examined according to the 2019 edition of the Chinese Society of Clinical Oncology (CSCO) guidelines and the 2013 edition of the American College of Radiology (ACR) Breast Imaging Reporting and Data System (BI-RADS). The patient was placed in the supine or lateral position, and the bilateral arms were abducted and raised to fully expose the bilateral breasts and axillae. The location, number, size, boundary, morphology, internal echo, calcification of lymph nodes, cortical thickness, aspect ratio (L/S), and resistance index (RI) were measure and recorded. The clear two-dimensional images were stored. Any one of the following conditions is considered abnormal lymph node status: (1) spherical shape and/or L/S enlargement, (2) disappearance of lymphatic hilum structure, (3) abnormal blood flow signal detected by color Doppler Adler grading, and (4) eccentric thickening and interruption of the lymph node cortex.

### 3.2. Ultrasonography-Guided Fine Needle Aspiration Cytology (FNAC) of the Lymph Nodes

In this study, 64 patients with positive lymph nodes underwent ultrasound-guided fine needle aspiration cytology. Before aspiration, ultrasound positioning was performed to determine the aspiration point, and a 10 ml empty needle was injected into the suspected positive lymph node under ultrasound guidance. To confirm whether the needle tip is located in the suspicious lymph node, several times of advancing, retreating, and rotating were performed to obtain enough tissue. The aspirated tissue was smear for cytology.

### 3.3. Lymph Node Biopsy

All the included patients underwent sentinel lymph node biopsy. The sentinel lymph nodes were detected using the dye and radionuclide double tracer method based on the following surgical guidelines: (1) the radionuclide tracer is 99mTc-labeled sulfur colloid, which is injected into the gland layer in the areola region 6 and 9 o'clock point of the affected side within 3-18 hours before the surgery. Ultrasound guidance was applied to ensure the injection depth during injection. Both sides are injected in the same way. (2) The blue dye is methylene blue, which is injected 10-15 minutes before surgery around the tumor or in the areola of the affected side. During surgery, the sentinel lymph node biopsy was performed first, and the first blue-stained lymph node entered by the blue-stained lymphatic vessel was searched at the level of the anterior axillary line. Then, using the radionuclide method, the *γ* detector was used to search for the radionuclide-concentrated lymph nodes in the axilla. All lymph nodes that exceeded the maximum lymph node count by 10% are sentinel lymph nodes. After the sentinel lymph nodes were detected by the dye and radionuclide double tracer, the axilla was palpated. Surgical specimens were sent for routine pathological examination. The postoperative sentinel lymph node and axillary lymph node biopsy results were taken as the gold standard to calculate the true and false positive rate of axillary ultrasonography, FNAC, and both methods combined.

### 3.4. Statistical Analysis

Statistical analysis was performed using SPSS 22.0 and GraphPad Prism 6. The count data between groups were compared by the c2 test or Fisher's exact probability method. The statistically significant differences were analyzed by multivariate logistic regression analysis, and the ROC curve of the relevant indicators to predict the status of axillary lymph nodes was drawn. *P* < 0.05 was considered to be of statistical significance.

## 4. Result

### 4.1. Ultrasound Signs for Axillary Lymph Node Status

**Figure 1:**
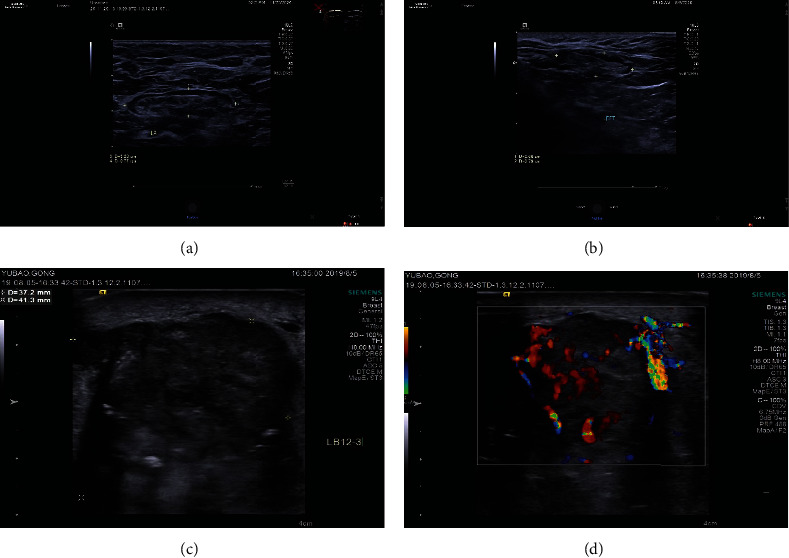
Ultrasound signs for different axillary lymph node statuses.

The negative ultrasound sign of lymph nodes was those with no obvious abnormal lymph node images as shown in Figures 1(a) and 1(b). Ultrasound described as a 20 mm^∗^10 mm hypoechoic mass in the axilla with clear boundary and regular shape and a wide door-like structure inside with no obvious calcification and no change in the posterior echo. The blood flow was graded as grade 0. No obvious signs of metastasis were seen.

The positive lymph nodes are shown in Figures 1(c) and 1(d). Ultrasound described as 20^∗^27, 8^∗^7 mm^∗^mm hypoechoic mass in the axilla, with irregular shape, unclear boundary, and no door-like structure. The blood flow grade was grade III.

### 4.2. Comparison of Ultrasound and FNAC for Assessment of Axillary Lymph Node Status

The true positive rate of ultrasound assessment of axillary lymph node status was 91.94%, which was higher than that of FNAC (85.96%); the false negative rate of ultrasound assessment of axillary lymph node status was 8.06%, lower than that of cell biopsy (14.04%). The false positive rate of ultrasound assessment of axillary lymph node status was 7.00%, which was higher than that of FNAC (0.00%) (Table 2).

### 4.3. Correlation between Clinicopathological Features and Axillary Lymph Node Metastasis

The lymph node positive rate was higher in patients with tumor size (2 mm < *T* ≤ 5 mm) and tumor stage III. The difference between groups was statistically significant (*P* < 0.05). There was no significant difference in age, family history, histological type, and Ki67 proliferation index between groups (*P* > 0.05) (Table 3).

### 4.4. Correlation of Ultrasound Signs with Axillary Lymph Node Metastases

The patients with cortical thickness ≥ 3, blood flow grades II-III, L/S ≥ 2, and RI ≥ 0.7 had higher lymph node positive rate. The difference between groups was statistically significant (*P* < 0.05). There was no significant difference between the other groups (*P* > 0.05) (Table 4).

### 4.5. Logistic Regression Analysis

**Figure 2:**
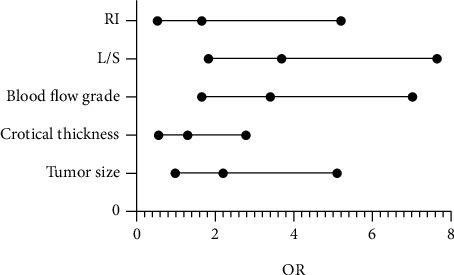
Logistic regression result.

The blood flow status and L/S of axillary lymph nodes were independent influencing factors of TNB axillary lymph node metastasis (*P* < 0.05). For patients with blood flow grades II-III, the risk of axillary lymph node (+) is 3.378 times that of blood flow grades 0-I; in patients with L/S ≥ 2, the risk of axillary lymph node (+) was 3.692 times that of L/S < 2 (Table 5 and Figure 2).

### 4.6. ROC Curve

**Figure 3:**
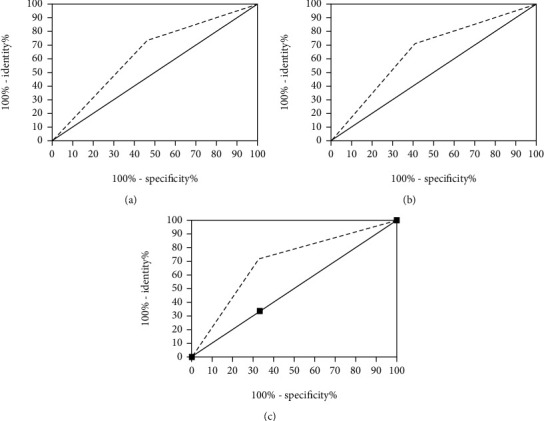
Axillary lymph node blood flow grade and L/S for predicting the status of axillary lymph nodes separately and combined.

The AUC of axillary lymph node blood flow grade and L/S for predicting the status of axillary lymph nodes were 0.6329 and 0.6498, respectively, and the AUC of the combined prediction was 0.6898 (Table 6, Figure 3).

## 5. Discussion

Axillary lymph nodes are the most common metastatic site of breast cancer. The status of axillary lymph nodes is often used to guide the treatment and prognosis of breast cancer patients before surgery. TNBC is a highly heterogeneous breast tumor. Compared with other subtypes of breast cancer, TNBC is more sensitive to chemotherapy, but has strong invasiveness and rapid progression, and is more prone to lymph node metastasis. Some studies have reported that more than 30% of TNBC patients have positive axillary lymph nodes [9]. Therefore, it is very important to accurately assess the status of axillary lymph nodes in TNBC patients. Currently, the status of axillary lymph nodes in patients with TNBC can be assessed by imaging examinations, FNAC, and sentinel lymph node biopsy. Axillary lymph node dissection can also clarify the status of lymph nodes, but it will bring complications such as upper limb edema, and it has been gradually replaced by sentinel lymph node biopsy. Yet, sentinel lymph node biopsy still has a certain false negative rate, and its standard includes radionuclide imaging, which is high cost and requires radiation.

With the rapid development of imaging-related technologies, imaging-assisted examination including ultrasound, mammography, computerized tomography, and magnetic resonance imaging is widely used in the diagnosis of axillary lymph node metastasis. The above several imaging-assisted examination methods have their own advantages and disadvantages. Compared with other methods, ultrasound is recommended by many researchers because of its high diagnostic rate, noninvasiveness, lower cost, easy operation, and real-time detection [3, 10, 11]. At present, many studies evaluated the relationship between the axillary lymph node ultrasound characteristics and the properties of axillary lymph nodes. Some research reported that the L/S of axillary lymph nodes is of great significance for judging metastasis of axillary lymph nodes [12, 13]. Some researchers believe that disappearance of lymph node hilum is helpful for judging metastasis of lymph nodes. Additionally, the thickened lymph node cortex and the change of vascular pattern of the lymph node are both critical sign lymph node metastasis. Previous studies using ultrasound signs to predict axillary lymph node in breast cancer patients reported that the sensitivity of axillary ultrasound can reach 76%, and the negative prediction rate can reach 89% [14]. Du et al. [15] reported that the morphology and blood flow grading of axillary lymph nodes in ultrasound signs were independent influencing factors for predicting lymph node metastasis. Luo et al. [12] constructed an ROC curve for predicting axillary lymph node status using ultrasound signs (lymph node size, lesion boundary). Its AUC reached 0.75 (95% CI: 0.70-0.79) and 0.91 (95% CI: 0.84-0.97), indicating a reliable predictive ability. Thus, analyzing the correlation between ultrasound features and axillary lymph node metastatic status and developing a predictive model based on these features will have clinical values.

This study focused on the relationship between the diagnosis of breast cancer axillary lymph node metastases and its characteristics of ultrasonography. There are some limitations of this study. For example, there is no specific distinction between the conditions of axillary lymph node ultrasonography. The results might be biased due to different sonographers and different instrument resolutions. In addition, this study is a retrospective study, and it is difficult to collect complete clinical data of patients. These factors may have an impact on the results, and further studies are warranted.

In conclusion, the characteristics of ultrasound images combined with clinicopathological characteristics can predict the metastasis of axillary lymph nodes in TNBC patients with high prediction efficiency. Preoperative ultrasound signs could help clinical decisions for axillary lymph node surgery.

## Figures and Tables

**Table 1:** Baseline information of the patients.

Subject	*n*	%
Age (years)		
<48	82	50.62
≥48	80	49.38
Family history of breast cancer		
Yes	11	6.79
No	151	93.21
Tumor size		
*T*_1_ (*T* < 2)	25	15.43
*T*_2_ (2 < *T* ≤ 5)	104	64.20
*T*_3_ (5 < *T*)	33	20.37
Axillary lymph node status		
Positive	62	38.27
Negative	100	61.73
Histology		
Ductal	143	88.27
Lobular	12	7.41
Others	7	4.32
Ki67		
≤14	14	8.64
>14	148	91.36

**Table 2:** Accuracy of different methods assessing the axillary lymph node status.

Method	*N*	True positive rate (%)	False positive rate (%)	False negative rate (%)
Ultrasound	162	91.94	7.00	8.06
FNAC	64	85.96	0.00	14.04

**Table 3:** Correlation between clinicopathological features and axillary lymph node metastasis.

Subject	Axillary lymph node status			
	Positive (*N* = 62)	Negative (*N* = 100)	*χ* ^2^	*P*
Age (years)				
<48	33	49	0.2734	0.6011
≥48	29	51		
Family history of breast cancer				
Yes	2	9	2.016	0.1556
No	60	91		
Tumor size				
*T*_1_ (*T* < 2 mm)	1	24	29.33	<0.0001
*T*_2_ (2mm < *T* ≤ 5 mm)	37	67		
*T*_3_ (5mm < *T*)	24	9		
Histology				
Ductal	58	85	2.916	0.2327
Lobular	3	9		
Others	1	6		
Ki67				
≤14	3	11	1.840	0.1749
>14	59	89		

**Table 4:** Correlation between ultrasound signs and axillary lymph node metastasis.

Subject	Axillary lymph node status			
	Positive (*N* = 62)	Negative (*N* = 100)	*χ* ^2^	*P*
Quadrant				
Upper-outside	32	52	1.287	0.8637
Lower-outside	11	17		
Lower-inside	2	5		
Lower-outside	10	18		
Other	8	8		
Cortical thickness (mm)				
<2	11	34	5.290	0.0214
≥2	52	66		
Internal echo				
Homogeneous	9	12	0.2148	0.6431
Heterogeneous	53	88		
Margin				
Smooth	14	34	2.916	0.2327
Not smooth	48	66		
Shape				
Regular	8	21	0.2908	0.5897
Irregular	54	79		
Parallel to the skin				
Yes	34	57	0.0726	0.7876
No	28	43		
Calcification				
Yes	25	35	0.4649	0.4953
No	37	65		
Blood flow grade				
0-I	17	64	20.49	<0.0001
II-III	45	36		
Posterior				
No change	39	69	2.897	0.4078
Deterioration	6	14		
Enhancement	9	8		
Mixed	8	9		
L/S				
<2	18	59	13.78	0.0002
≥2	44	41		
RI				
<0.7	51	94	5.618	0.0178
≥0.7	11	6		

**Table 5:** Logistic regression analysis.

Subject	*B* (beta, mean regression coefficient)	Std. error	Wald (chi-square value)	Degree of freedom (df)	*P*	OR
Tumor size	0.7927	0.4240	3.496	1	0.0615	2.209
Cortical thickness	0.2532	0.3815	0.4406	1	0.5068	1.288
Blood flow grade	1.217	0.3730	10.65	1	0.0011	3.378
L/S	1.306	0.3674	12.64	1	<0.0001	3.692
RI	0.5007	0.5798	0.7456	1	0.3879	1.6498

**Table 6:** ROC curve-related parameters of each index predicting axillary lymph node status.

Subject	AUC	Std. error	95% CI	*P*
Blood flow grade	0.6329	0.0446	0.5455-0.7203	0.0045
L/S	0.6498	0.0442	0.5631-0.7366	0.0014
Combination	0.6898	0.0431	0.6053-0.7744	<0.0001

## Data Availability

Emails could be sent to the address below to obtain the shared data: guwenping2008@163.com.
